# Skin-Based α-Synuclein Deposits Detection Across the Prodromal Continuum of Synucleinopathies: Updated Evidence and Perspectives

**DOI:** 10.3390/biom16030376

**Published:** 2026-03-02

**Authors:** Seyed-Mohammad Fereshtehnejad

**Affiliations:** 1 Pacific Parkinson’s Research Centre (PPRC), Djavad Mowafaghian Centre for Brain Health, University of British Columbia, Vancouver, BC V6T 1Z4, Canada; sm.fereshtehnejad@ki.se; 2 Division of Neurology, Faculty of Medicine, University of British Columbia, Vancouver, BC V6T 1Z4, Canada; 3 Division of Clinical Geriatrics, Department of Neurobiology, Care Sciences and Society (NVS), Karolinska Institutet, 171 77 Stockholm, Sweden

**Keywords:** Parkinson’s disease, dementia with Lewy bodies, synucleinopathy, REM sleep behavior disorder, pure autonomic failure, skin biopsy, phosphorylated α-synuclein, α-synuclein seed amplification assay

## Abstract

Parkinson’s disease (PD) and associated synucleinopathies are preceded by a prolonged prodromal phase during which neurodegenerative processes evolve years before the onset of motor or cognitive symptoms. Identifying biologically specific and accessible biomarkers during this window is critical for early diagnosis, risk stratification, and the development of disease-modifying therapies. Increasing evidence supports the skin as a key peripheral tissue involved in synucleinopathy, offering a minimally invasive source for in vivo detection of pathological α-synuclein. This review summarizes current evidence on skin-derived biomarkers across the prodromal continuum of PD, with particular emphasis on skin biopsy-based detection of phosphorylated α-synuclein and α-synuclein seed amplification assays (SAAs). Findings in high-risk prodromal phenotypes, including idiopathic REM sleep behavior disorder (iRBD) and pure autonomic failure (PAF), are critically reviewed. Emerging data suggest that cutaneous α-synuclein pathology may precede nigrostriatal dopaminergic degeneration and may predict phenoconversion to overt synucleinopathies. Important knowledge gaps are highlighted, including the lack of data in other prodromal phenotypes such as hyposmia. Overall, skin-based biomarkers appear to represent promising, scalable tools for biological diagnosis, prognostication, and enrichment of prodromal PD cohorts in clinical trials.

## 1. Introduction

Synucleinopathies are increasingly conceptualized as systemic disorders characterized by the misfolding and propagation of α-synuclein across central and peripheral nervous systems, preceded by a prolonged prodromal phase that may span decades. Parkinson’s disease (PD) represents one major phenotypic outcome within this broader spectrum, alongside dementia with Lewy bodies (DLB) and multiple system atrophy (MSA). PD is one of the fastest growing neurological disorders worldwide, with incident and prevalent cases more than tripling over recent decades [1,2] and projections indicating continued rises in burden through 2050 [3]. PD is a gradually progressive neurodegenerative disorder with a remarkable heterogeneity in clinical manifestations and many unknowns in its pathogenesis [4]. PD is conventionally defined by its cardinal motor features (i.e., bradykinesia, rigidity, tremor, postural instability); nevertheless, there is a large list of non-motor manifestations often preceding motor signs by years and causing substantial morbidity throughout the disease course. For the past half century, the mainstay of symptomatic treatment for motor symptoms has been dopaminergic therapy. However, disease-modifying therapies remain elusive, underscoring the critical need for tools and/or markers that enable much earlier and biologically grounded diagnosis. Mounting epidemiological and longitudinal data suggest that PD is preceded by a prolonged prodromal phase, often extending 10–20 years or more before motor (in PD) or cognitive onset (in DLB), during which non-motor features and subclinical neurodegeneration gradually accumulate [5,6,7]. This prodromal window represents a golden period for intervention, during which disease-modifying trials might best target pathophysiological cascades before substantial and irreversible nigrostriatal and cortical damage occurs.

There is an urgent need for robust biomarkers that can (1) identify individuals at high risk for developing PD years before clinical diagnosis, (2) biologically define prodromal synucleinopathy, and (3) monitor disease progression and treatment response across the continuum from at-risk states to manifesting PD or DLB. While imaging, genetic, and fluid biomarkers have advanced [8,9], skin biopsy has emerged as a practical and pathologically specific tool to detect phosphorylated α-synuclein and related signatures of synucleinopathy in vivo, with encouraging sensitivity and specificity across the synucleinopathy spectrum [10]. The skin also offers access to peripheral autonomic and small nerve fibers and to cutaneous microbiota, providing a unique interface to study both neural and non-neural components of prodromal PD [11].

This review focuses on skin-derived biomarkers across the prodromal continuum of synucleinopathies, with a particular emphasis on skin biopsy-based measures. First, the concept of prodromal synucleinopathy and its clinico-pathological correlates is outlined. Subsequent sections review dermal nerves’ involvement in prodromal synucleinopathy, practical considerations of skin biopsy, and application of immunohistochemistry methods and seed amplification assays (SAAs) using skin biospecimens as a tool to detect misfolded α-synuclein during the prodromal stage, followed by dedicated discussions of the latest evidence on skin biopsy findings in idiopathic REM sleep behavior disorder (iRBD) and pure autonomic failure (PAF), two high-risk prodromal synucleinopathy phenotypes. The potential of cutaneous α-synuclein deposits as a prognostication tool for phenoconversion and progression is then explored.

## 2. Prodromal Phase of Synucleinopathies

The prodromal stage of PD spans at least two decades and is dominated by non-motor symptoms well before classic motor signs emerge [5,6]. Longitudinal iRBD cohort studies show that smell and taste loss [6], sexual dysfunction, and upper gastrointestinal symptoms such as reflux and gastroparesis [12] can begin around 20 years before clinical diagnosis, with measurable increases already 15–17 years pre-diagnosis [5,12]. In a Montreal RBD cohort with nearly 10–15 years of prospective follow-up, hyposmia was found to be the earliest quantified marker (predicted onset ≈ 20–22 years before phenoconversion), followed by impaired color vision and autonomic features including constipation, orthostatic hypotension, and erectile dysfunction starting roughly 10–16 years before diagnosis, and later urinary dysfunction around 6–7 years before [5,6]. Large amounts of data from medical records of the United States Veterans Affairs healthcare system further suggest that constipation, urinary symptoms, and certain skin conditions (for example dermatophytosis) can be prodromal for more than a decade, reinforcing that gut, genitourinary, and skin involvement are early systemic manifestations [12]. Subtle cognitive decline and mood/behavioral changes typically appear 5–9 years before diagnosis, while mild motor complaints (changes in handwriting, turning in bed, walking, speech, and facial expression) arise about 7–11 years prior but remain modest until close to conversion; objective motor signs on examination and quantitative motor tests become clearly abnormal about 5–10 years before diagnosis and then accelerate over the last 3–4 years, with bradykinesia preceding rigidity and tremor [6]. Even from the prodromal stage, we overtly note the heterogeneity in clinical manifestations, supporting the existence of various pathological subtypes early on [13].

In iRBD cohorts, most phenoconversions are to Lewy body diseases, with PD and DLB accounting for the majority and MSA for the minority. In the original Montreal iRBD cohort, about half developed a parkinsonism-first syndrome (mostly PD) and about half a dementia-first syndrome meeting criteria for DLB (PD ≈ 45%, DLB ≈ 47%, and MSA ≈ 7–8%) [6]. In the updated extended cohort followed up to 14 years before diagnosis, roughly similar proportions of PD and DLB (each ≈ 45–50%) and a small MSA fraction (≈6%) were documented as the synucleinopathy phenotypes among the phenoconvertors [5]. These data align with larger multicenter iRBD studies showing that more than 80% of patients develop a manifest synucleinopathy within about 10–15 years, with PD and DLB being the predominant outcomes and MSA consistently rare but clinically important [14,15].

## 3. Skin Involvement in Synucleinopathies

The skin is increasingly recognized as a key peripheral organ involved in PD pathogenesis, indicating early autonomic dysfunction and α-synuclein pathology [16]. Multiple dermatologic conditions including melanoma, seborrheic dermatitis, sweating disorders, bullous pemphigoid, rosacea, perioral dermatitis, and peripheral neuropathy-related skin manifestations have been reported to co-occur more frequently in individuals with PD [16,17,18]. Abnormal phosphorylated α-synuclein aggregates have been identified in dermal nerve fibers, especially in regions associated with sweat glands, muscle fibers, and blood vessels [19]. This underscores the skin as a pathway to investigating synucleinopathy. Although skin serves as an accessible peripheral source for α-synuclein detection, the biological origin of aggregated α-synuclein in dermal homogenates remains undetermined. The origins of these seeds are speculated to include cutaneous nerve terminals, Schwann cells, or fibroblasts, but evidence remains ambiguous [20]. Regardless of the source, at present, there is no evidence to suggest that skin microbiota directly influences the integrity, aggregation state, or seeding properties of α-synuclein detected in skin biopsies.

## 4. Feasibility and Practical Aspects of Skin Biopsy

**Figure 1:**
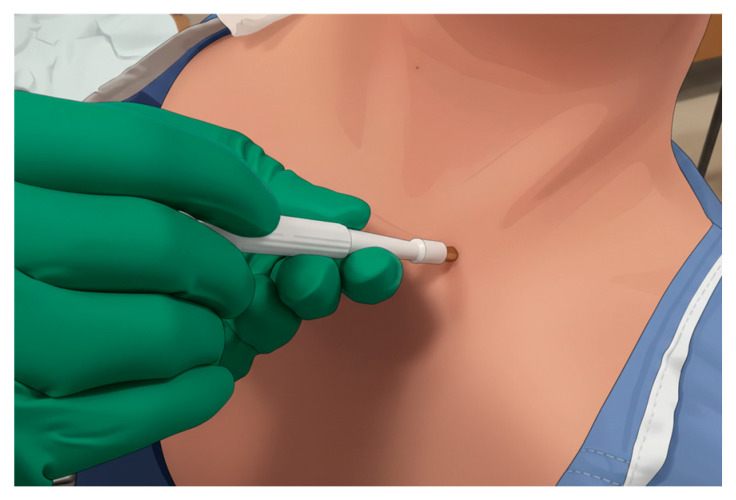
Skin punch biopsy procedure at C8 dermatome level.

Skin biopsy has emerged as a minimally invasive and well-tolerated procedure for detecting phosphorylated α-synuclein in peripheral nerve fibers [21]. Skin biopsy has practical advantages over other diagnostic approaches for synucleinopathies such as lumbar puncture. The procedure typically involves obtaining 3 mm punch biopsies from multiple anatomical sites, most commonly the cervical C7 or C8 paravertebral area (Figure 1), thigh (15 cm above the patella), and distal leg (10 cm above the lateral malleolus), with two samples often taken at each location (right and left) to improve detection rates [22]. The skin is the most accessible peripheral tissue for both single and repetitive sampling, and compared to most other organ biopsies, cutaneous biopsy is considerably easier to perform and better tolerated by patients. Additionally, skin biopsy is less expensive and more widely accessible than imaging modalities, such as dopamine transporter single-photon emission computed tomography (DaTscan), and has demonstrated high inter- and intra-laboratory reproducibility. Recent clinical utility studies have shown that synuclein skin biopsy influenced diagnosis or management in up to 78% of cases with diagnostic uncertainty [23]. The development of high-throughput techniques such as real-time quaking-induced conversion (RT-QuIC), which exploits the prion-like seeding activity of α-synuclein, offers a promising avenue to streamline analysis and reduce examiner-dependent variability, although larger validation studies are still needed to standardize the SAA protocols on biosamples including skin biopsies.

## 5. Methods to Detect Alpha-Synuclein Deposits in Skin Biopsies

The primary methods used to detect α-synuclein deposits in skin biopsies are immunofluorescence/immunohistochemistry for phosphorylated α-synuclein and SAAs with either real-time quaking-induced conversion (RT-QuIC) or protein misfolding cyclic amplification (PMCA). Both techniques have demonstrated high diagnostic accuracy, though they detect different aspects of α-synuclein pathology and have distinct technical requirements (Table 1).

In immunofluorescence or immunohistochemistry techniques, the procedure typically involves obtaining 3 mm biopsies from selected hairy skin sites, most commonly the cervical paravertebral region (C8), distal thigh, and distal leg. Among these sites, the cervical region consistently demonstrates the highest diagnostic sensitivity [24]. Studies show that sampling two cervical sites significantly improves detection rates compared with a single biopsy, while combining cervical and distal leg sites yields comparable sensitivity [24]. Sampling multiple anatomical regions is therefore recommended, as cutaneous α-synuclein deposition displays regional variability. Following collection, tissue samples are fixed using paraformaldehyde–lysine–periodate and sectioned at a thickness of 50 μm [25]. Section thickness is a critical determinant of assay performance. Compared with thinner sections, 50 μm sections preserve the three-dimensional architecture of dermal nerve fibers and autonomic structures, substantially increasing the likelihood of detecting intraneural pS129-α-synuclein deposits, particularly around sweat glands, arrector pili muscles, and dermal blood vessels. Antigen retrieval is the next pivotal step in the assay. Application of proteinase K has emerged as the preferred method, as it significantly increases detection rates compared to standard protocols or formic acid treatment [26]. Without optimized antigen retrieval, pathological aggregates may remain inaccessible to antibody binding, leading to reduced sensitivity. Dual or co-immunostaining is performed using antibodies targeting pS129-α-synuclein and a pan-axonal marker, most commonly protein gene product 9.5. This approach enables precise localization of α-synuclein deposits within dermal nerve fibers rather than surrounding connective tissue [25]. Antibody selection is a major technical consideration. Among available monoclonal antibodies, the D1R1R clone, when combined with proteinase K treatment, demonstrates superior sensitivity and specificity compared with commonly used alternatives [26]. Imaging is performed using confocal microscopy, which provides high-resolution, three-dimensional visualization of nerve fibers and α-synuclein aggregates. Computer-aided image analysis quantifies pS129-α-synuclein immunopositivity in dermal nerve fibers, with results normalized to nerve fiber density to account for small-fiber neuropathy that may coexist in PD patients. This normalization is critical because reduced nerve fiber density could artificially lower the apparent pS129-α-synuclein deposition if not accounted for [25]. Colocalization of pS129-α-synuclein signals appears as yellow fluorescence in merged images, confirming intraneural deposition. Immunofluorescence shows high reproducibility between neighboring skin samples and achieves sensitivities of 72–92.7% and specificities of 90–100% for distinguishing synucleinopathies from controls [25,27]. However, inter-laboratory variability remains a significant challenge across different centers [25]. This variability is driven by methodological differences including biopsy site selection, tissue section thickness, fixation methods, antibody clones, and image acquisition and processing protocols.

SAAs have been developed based on the fundamental pathological property that misfolded α-synuclein aggregates behave like prions. They can template and propagate their abnormal conformation to normal α-synuclein proteins. When even minute amounts of pathological α-synuclein “seeds” from patient samples are introduced into a reaction containing recombinant α-synuclein monomers, these seeds catalyze the misfolding and aggregation of the monomers into fibrils through a cyclical amplification process [28,29]. RT-QuIC and PMCA are the two main seed amplification assay platforms, both adapted from prion detection methods originally developed for Creutzfeldt–Jakob disease. These assays detect misfolded α-synuclein by exploiting its ability to template the conversion of normal recombinant α-synuclein into pathological aggregates. RT-QuIC uses repeated shaking to fragment existing α-synuclein aggregates in biological samples such as CSF, skin, olfactory mucosa, or saliva, generating smaller seeds that accelerate misfolding of the substrate; it is generally faster and highly sensitive, though slightly less specific than PMCA [28,30]. PMCA relies on cycles of incubation and sonication, with sonication providing more aggressive fragmentation and exponential amplification of seeding activity [31]. In both platforms, biological samples are mixed with recombinant α-synuclein and thioflavin T, a fluorescent dye that binds amyloid fibrils. Cycles of agitation and rest promote seed generation and fibril growth, while fluorescence is monitored in real time as fibrils accumulate. A predefined fluorescence threshold is used to define assay positivity, reflecting successful amplification of pathological seeds. Despite strong diagnostic performance, some inter-laboratory variability persists, driven by differences in recombinant protein preparation, thioflavin T concentration, buffer composition, amplification conditions, and positivity cutoffs, highlighting the need for protocol standardization [28].

**Table 1:** Comparative methodological considerations for skin-based detection of α-synuclein.

	Immunofluorescence/ Immunohistochemistry	Seed Amplification Assay
Biological Target	Phosphorylated α-synuclein (pSer129) aggregates	α-synuclein seeding activity
Primary Readout	Microscopic visualization of intra-axonal deposits	Fluorescence kinetic curve (lag time, max amplitude)
Operator Dependence	High (manual staining, interpretation variability)	Low–Moderate (largely automated once standardized)
Inter-Laboratory Variability	High (tissue thickness, antibody selection, fixation method)	High (concentrations, buffers, timing)
Quantitative Output	Semi-quantitative	Kinetic metrics (e.g., slope, lag time)
Diagnostic Accuracy (head-to-head comparison [27])	Very high (97%)	High (82%)

Alpha-synuclein SAAs have demonstrated excellent diagnostic accuracy for detecting PD and other synucleinopathies, with pooled sensitivities of 86–96% and specificities of 93–100% when distinguishing PD from healthy controls [32]. Among all biospecimens tested with α-synuclein SAAs, skin shows the highest sensitivity (92%) and performs comparably to CSF and remarkably better than olfactory mucosa [33,34]. Individual studies report sensitivity between 80 and 96% and specificities between 90 and 100% for skin SAAs in synucleinopathies [25]. A validation study showed that skin-based RT-QuIC achieved near-perfect discrimination between PD patients and controls, with robust differentiation between synucleinopathies (PD, MSA, DLB) and non-synucleinopathies (tauopathies, controls) [28]. A comparative study concluded that both immunofluorescence and RT-QuIC revealed good diagnostic accuracy; however, immunofluorescence demonstrated superior value and optimal reproducibility [27].

## 6. Skin Biopsy in Idiopathic REM Sleep Behavior Disorder (iRBD)

Isolated REM sleep behavior disorder (iRBD) has emerged as the most specific prodromal manifestation of α-synucleinopathy, characterized by a loss of normal REM sleep atonia leading to dream-enacting behaviors [35]. Longitudinal studies have conclusively demonstrated that more than 80% of individuals with polysomnography-confirmed iRBD will eventually phenoconvert to PD, DLB, or MSA, with phenoconversion rates approximating at 6% per year and exceeding 90% at extended follow-up [36,37]. Indeed, it has been shown that more than 95% of patients with iRBD will progress to a defined α-synucleinopathy within 14 years of diagnosis [38,39]. This remarkably high conversion rate distinguishes iRBD from other prodromal markers such as hyposmia or constipation, which, although sensitive, lack the same degree of specificity for synucleinopathy.

Alpha-synuclein deposition has been observed not only in manifest PD but also in preclinical motor/cognitive stages like iRBD, though data are more limited for skin than for CSF. Studies demonstrate high positivity rates in patients with iRBD and other at-risk populations across all tested biospecimens [40]. The largest and most comprehensive studies report CSF α-synuclein SAA positivity rates of 90% in iRBD, with a sensitivity of 90% and a specificity of 90% when distinguishing iRBD from healthy controls [41]. A meta-analysis found overall CSF positivity of 80% (95% CI 68–88%), while individual studies report ranges from 64% to 93% likely due to variability in cohort characteristics and timing of recruitment [42]. In iRBD populations, skin biopsy has demonstrated comparable performance to CSF, with a meta-analysis showing 74.8% positivity (95% CI 53.2–88.5%) for α-synuclein SAAs and 78.5% positivity (95% CI 70.4–84.9%) for phosphorylated α-synuclein immunofluorescence [42]. Findings from original studies evaluating the rate of α-synuclein detection in skin samples within various prodromal cohorts are listed in Table 2. The Barcelona study directly comparing skin and CSF found that skin punch biopsy and lumbar puncture have comparable mild adverse effects, tolerance, and acceptance, with 83% and 80% of participants stating that they would accept to undergo skin biopsy and lumbar puncture again for research purposes, respectively [43]. Another study was able to detect phosphorylated α-synuclein in 55.6% of individuals with iRBD [44]. Notably, the probability of phosphorylated α-synuclein positivity was higher in individuals with olfactory dysfunction, while the association with diminished dopamine transporter SPECT ligand density was weaker, suggesting that skin biopsy positivity may occur in patients with isolated RBD and normal dopamine transporter SPECT, at least two years prior to nigrostriatal degeneration [44,45,46]. Detection of α-synuclein deposits in skin could also assist in differentiating iRBD as a prodromal stage of neurodegenerative parkinsonism from secondary RBD due to narcolepsy [47]. Using unilateral biopsies at C8 and the distal leg, one study revealed phosphorylated α-synuclein deposits in 87% of patients with iRBD but in none of the individuals with RBD secondary to type 1 narcolepsy [47].

## 7. Skin Biopsy in Pure Autonomic Failure (PAF)

Pure autonomic failure (PAF) is defined by chronic neurogenic orthostatic hypotension and diffuse autonomic failure in the absence of overt parkinsonism or dementia. PAF is a highly significant non-motor prodromal feature of synucleinopathy, with approximately 33–35% of patients phenoconverting to more widespread neurodegenerative synucleinopathies over time, most commonly to PD, DLB, or MSA [56,57,58]. Multiple studies have shown high levels of phosphorylated α-synuclein deposition in cutaneous autonomic fibers in patients with PAF (Table 2). Donadio et al. first established that skin sympathetic fibers with phosphorylated α-synuclein were found in all PAF patients while absent in controls, suggesting the potential of phosphorylated α-synuclein-positive fibers as a biomarker for PAF [11]. A larger study confirmed that misfolded phosphorylated α-synuclein was detected in 100% of PAF patients and not in healthy controls or non-synucleinopathic autonomic neuropathies, offering high specificity [53,54]. In contrast to PD, PAF exhibited uniform phosphorylated α-synuclein positivity across all sampled sites, while PD appears to have a length-dependent pattern [59]. Further research included mixed synucleinopathy cohorts and demonstrated high sensitivity and specificity for p-syn in PAF [55].

## 8. Skin Biopsy in Other Prodromal Phenotypes

While high-risk prodromal phenotypes such as iRBD and PAF provide strong biological enrichment for underlying synucleinopathy, other putative prodromal states, including isolated hyposmia and constipation-predominant presentations, are less specific and considerably more prevalent in the general population. This reduced specificity poses practical challenges in identifying individuals with true prodromal synucleinopathy based solely on these features, thereby limiting their current utility for biomarker validation studies. Nevertheless, emerging evidence demonstrates that CSF α-synuclein SAAs can detect seeding activity in hyposmia-enriched prodromal cohorts [25]. Even in iRBD cohorts, the percentage of dermal structures innervated by phospho-α-synuclein-positive fibers showed significant correlation with olfactory function, suggesting that the extent of peripheral α-synuclein pathology reflects olfactory dysfunction severity [45]. These findings provide a strong rationale for future investigations evaluating less invasive approaches such as skin punch biopsy-based detection methods in broader prodromal populations, particularly the hyposmia phenotype.

## 9. Skin α-Synuclein Deposition as Prognostication Tool

There is emerging evidence supporting the use of skin α-synuclein deposits as a prognostic tool to predict phenoconversion to overt synucleinopathy during the prodromal stage of parkinsonism. In a prospective study, 26% of patients with iRBD and positive phosphorylated α-synuclein skin biopsy phenoconverted to clinical Parkinson’s disease within just 3 years of follow-up [49]. This represents a substantially higher and more rapid conversion rate compared to the general iRBD population. A prospective multicenter study of 151 patients with early-stage parkinsonism (<18 months duration) demonstrated that skin intraneural phosphorylated α-synuclein was positive at baseline in 30 of 44 patients who were not diagnosed with PD until follow-up, indicating that the biomarker detected pathology years before diagnostic criteria were met [60]. The assay showed remarkable temporal stability, with 96% (42 of 44) of tested patients showing the same results at baseline and 18-month follow-up [60]. Importantly, skin biopsy positivity can be found in iRBD patients with normal dopamine transporter SPECT at least 2 years before nigrostriatal decline [45]. This suggests that peripheral α-synuclein deposition may precede central dopaminergic neurodegeneration, as detected by SPECT, making it a potentially earlier prognostic marker than neuroimaging.

## 10. Discussion and Conclusions

Skin biopsy shows great promise as an in vivo diagnostic biomarker for prodromal neurodegenerative synucleinopathy. Accumulating data indicate that skin biopsy is a viable, minimally invasive, and biologically specific biomarker for identifying α-synuclein pathology throughout the prodromal phase of PD and other synucleinopathies. The immunohistochemical identification of phosphorylated α-synuclein and seed amplification assays conducted on skin samples reveal high diagnostic precision, reproducibility, and clinical applicability, especially among high-risk prodromal phenotypes like iRBD and PAF. Although the specificity of skin-derived α-synuclein detection appears high in research settings, real-world implementation may introduce potential sources of diagnostic uncertainty. CSF α-synuclein SAAs demonstrate 4–5% false positivity among healthy controls but 10–24% in disease mimics and other neurological disorders [25], while skin SAAs show 0–10% false positivity in healthy controls [34,61]. Given the prolonged preclinical phase of synucleinopathies, some apparent “false positives” in cross-sectional analyses may instead represent non-manifesting or pre-prodromal synucleinopathy. Skin α-synuclein pathology seems to manifest quite early, frequently preceding the reduction in nigrostriatal dopaminergic function, and holds potential as a prognostic indicator for earlier phenoconversion. Despite this potential, several challenges hinder its routine clinical application. There is a need for standardization in biopsy site selection, tissue processing and assays protocols, and inter-laboratory quality control to ensure reproducibility. Additionally, cost-effectiveness analyses are required to assess its value compared to current traditional methods like dopaminergic imaging. Regulatory approval will necessitate multicenter validation studies to establish clinical utility, especially in prodromal populations for trial stratification. As disease-modifying trials aimed at changing at-risk individuals during prodromal stages, standardized, scalable, and validated skin-based biomarkers may be pivotal in risk classification, biological diagnosis, and outcome evaluation. Future longitudinal studies that incorporate skin biomarkers alongside imaging, fluid, genetic, and digital markers will be essential to delineate their optimal use in precision preventive and early intervention techniques for Parkinson’s disease. Moreover, evidence on skin biopsy α-synuclein deposits or SAA findings in other prodromal phenotypes, such as hyposmia, is currently lacking and warrants investigation in future studies.

## Figures and Tables

**Table 2:** Skin α-synuclein detection in individuals with various phenotypes of prodromal Parkinson’s disease and other synucleinopathies.

Prodromal Cohort	Study	Method	Positivity Rate
Idiopathic REM Sleep Behavior Disorder (iRBD)	Doppler et al. (2017) [44]	Immunofluorescence	55.6%
	Antelmi et al. (2017) [48]	Immunofluorescence	75.0%
	Antelmi et al. (2019) [47]	Immunofluorescence	86.7%
	Al-Qassabi et al. (2021) [49]	Automated Immunohistochemical Assay	82%
	Doppler et al. (2021) [46]	Immunofluorescence	75% (baseline) 79% (follow-up)
	Miglis et al. (2021) [50]	Immunohistochemical Assay	64%
	Iranzo et al. (2023) [43]	SAA (RT-QuIC)	76.9%
	Kuzkina et al. (2023) [51]	SAA (RT-QuIC) Immunohistochemical Assay	97.4%
	Liguori et al. (2023) [52]	SAA (RT-QuIC) Immunofluorescence	59.1% 78.0%
Pure Autonomic Failure (PAF)	Donadio et al. (2013) [11]	Immunofluorescence	100%
	Donadio et al. (2016) [53]	Immunofluorescence	100%
	Donadio et al. (2018) [54]	Immunofluorescence	100%
	Giannoccaro et al. (2020) [55]	Double Immunofluorescence	100%

## Data Availability

No new data were created for this review article.

## Abbreviations

The following abbreviations are used in this manuscript:

DLB	Dementia with Lewy bodies
PD	Parkinson’s disease
MSA	Multiple system atrophy
RBD	REM sleep behavior disorder
PAF	Pure autonomic failure
SAA	Seed amplification assay
SPECT	Single-photon emission computed tomography
CI	Confidence interval
